# Knox Co. Medical Society

**Published:** 1852-07

**Authors:** E. S. Cooper, L. C. Lane

**Affiliations:** President; Secretary


*Knox County Medical Society.*

PURSUANT to previous notice, there was a meeting of the "Knox County Medical Society" at Galesburg, June 26, 1852.

On motion, a committee of three, consisting of Drs. Cooper, Bunce and Spaulding, were appointed to draught a constitution and by-laws for said society.

The aforesaid committee, after deliberation, submitted the following, which the society approved and adopted, viz.:

ART. 1st. The Society shall be known and designated the *Knox County Medical Society*.

ART. 2d. The physicians present at the adoption of this constitution shall be members of the society.

ART. 3d. Any regular graduate of an orthodox school, or any physician passing a satisfactory examination before the board of censors, may become a member by a vote of the society.

ART. 4th. The officers of the society shall consist of a President, Vice President, Secretary, who shall also act as Treasurer, and three Censors,—to be elected annually by a vote of two-thirds of the society.

ART. 5th. The society shall be governed by the code of ethics adopted by the Illinois State Medical Society.

ART. 6th. This constitution may be altered or amended by a vote of two-thirds of the members of the society.

The society as organized under the aforesaid constitution consists of the following physicians, viz.:

E. S. Cooper, from St. Louis University, Mo.,

J. W. Spaulding, from Rush Medical College, Ill.,

J. Duncan, from University of Missouri, Mo.,

J. W. Brewer, from Ohio Medical Institution, O.,

S. C. Patterson, from Pennsylvania University,

R. C. Edgerton, from Rush Medical College, Ill.,

Jas. Bunce, from University of New York,

L. C. Lane, from Jefferson Medical College, Phil.

The society next proceeding to an election of officers, the following were chosen, viz.:

Jas. Bunce, President; J. W. Spaulding, Vice President; L. C. Lane, Secretary, and Drs. Duncan, Lane and Brewer, Censors.

On motion, Drs. Cooper and Spaulding were each appointed to read an original essay at the next regular meeting.

On motion, the secretary was instructed to forward a copy of the proceedings of this society for publication in the *North Western Med. and Surg. Journal*, and in the papers of this county.

On motion, the society adjourned to meet the first Saturday in September next, 10 o'clock A. M., at Knoxville court house.

E. S. COOPER, M.D., President.

L. C. LANE, M.D., Secretary.

